# Care-seeking behaviour of adolescents with patellofemoral pain: a retrospective cohort study

**DOI:** 10.12688/f1000research.75667.1

**Published:** 2022-02-09

**Authors:** Michael Skovdal Rathleff, Camilla Rams Rathleff, Jens Lykkegaard Olesen, Ewa M Roos, Sten Rasmussen, Alessandro Andreucci, Martin Bach Jensen

**Affiliations:** 1 Department of Physiotherapy and Occupational Therapy, Aalborg University Hospital, Aalborg, 9000, Denmark; 2 Center for General Practice at Aalborg University, Aalborg University, Aalborg Øst, 9220, Denmark; 3 Department of Health Science and Technology, Faculty of Medicine, Aalborg University, Aalborg Øst, 9220, Denmark; 4 Institute of Sports Science and Clinical Biomechanics, University of Southern Denmark, Odense, 5230, Denmark; 5 Department of Clinical Medicine, Aalborg University, Aalborg Øst, 9220, Denmark

**Keywords:** Adolescents, care-seeking, knee pain, patellofemoral pain, treatment

## Abstract

Aim: The aim of this study was to assess the care-seeking behaviour among adolescents with patellofemoral pain (PFP).

Methods: This retrospective study included data on 121 adolescents with PFP enrolled in a randomized controlled trial. A questionnaire was sent to the general practitioner (GP) of each adolescent, assessing information on the consultation dates for knee pain, potential diagnoses, and treatment provided.

Results: 106/121 adolescents had been in contact with their GP, and 95 medical records of adolescents were available. Of the 95 adolescents with available medical records 60 had consulted their GP for knee pain. The median number of contacts was 1.5 (range 1-7). The GPs initiated treatment for 48 of the 60 adolescents and in most cases it was information and advice (36/48) or pain medication to a minor extent (6/48). Out of the 60 adolescents who consulted their GP 26 were subsequently referred to different types of health care professionals, in 11 out of 26 to physiotherapy, but also to the department of rheumatology or orthopaedics.

Conclusions *:* 63% of adolescents diagnosed with PFP had previously consulted their GP due to knee pain. Several types of treatments were initiated by the GP, but most commonly advice and information were given. Standardized and evidence-based treatment guidelines for adolescent knee pain in general practice are needed

## List of abbreviations

APA2011: Adolescent Pain in Aalborg 2011

GP: General Practitioner

PFP: Patellofemoral pain

RCT: Randomized controlled trial

## Introduction

Musculoskeletal pain is experienced by up to 40% of adolescents ^1^^–^^3^ and is a common reason for consulting a general practitioner (GP), who is often the first healthcare professional involved in the management and treatment of pain. ^4^^–^^6^ The knee is one of the most prevalent regions of reported pain. ^2^^,^^7^^–^^9^ The prevalence of knee pain in adolescents is between 19 and 31% ^2^^,^^8^^,^^10^^,^^11^ with patellofemoral pain (PFP) being one of the most common knee conditions among adolescents, experienced by approximately 6-7% of them. ^6^^,^^12^^–^^14^ Patellofemoral pain is defined as pain in the peri- or retro-patellar area experienced during activities that involve bending of the knee (cycling, climbing stairs or similar activities). Issues associated with long-standing PFP include high pain intensity and low quality of life. Long standing PFP is also associated with higher risk of interrupting participation in sport activities. ^12^^,^^15^

There are a number of different treatment options for PFP. A meta-analysis from high quality trials documents a positive effect of exercise therapy. ^16^ Exercise therapy has been advocated as the cornerstone in treatment of patients with PFP. ^16^ A randomized controlled trial (RCT) showed that exercise therapy combined with patient education was more effective compared to patient education alone. ^13^^,^^17^ At 12 months only 29% were fully recovered in the randomised group with patient education alone while 38% were fully recovered in the randomised group with patient education combined with exercise therapy. The proportion of adolescents fully recovered was significantly lower than what have been reported in previous similar exercise studies on adults with PFP (62-81% of patients receiving exercise therapy). ^18^^,^^19^

On average, the adolescents in the RCT reported knee symptoms for more than three years at inclusion, which is longer than previous trials in adult patients with PFP. ^13^^,^^18^^–^^20^ A long symptom duration before initiation of treatment is associated with poorer outcome after treatment which may partially explain the lower effect of exercise therapy among adolescents. ^20^ The question is if these adolescents had previously contacted their GP due to their knee pain, and if so, which recommendation was provided by the GP. This is important as early treatment may improve the effect of exercise therapy among adolescents with PFP, ^21^^,^^22^ and exercise therapy can only be prescribed if the adolescents decide to seek medical care as the GP is gatekeeper in Denmark. The purpose of this study was therefore to retrospectively assess the care-seeking behaviour of a cohort of adolescents with PFP using patient records from their GP.

## Methods

### Design

The design was a retrospective cohort study. ^13^ Ethical approval was obtained from the local ethics committee in the North Denmark Region (N-20110020) and the ethics committee approved that adolescents >15 years of age could consent on their own. All participants were required to give written informed consent for the researchers to use their data to explore the care pathway.

### Recruitment of participants

Adolescents with PFP were recruited from a population-based cohort (Adolescent Pain in Aalborg (APA) 2011, the APA2011-cohort) which included 2200 adolescents between 15-19 years. ^17^ Of the 2200 adolescents who answered the questionnaire, 724 reported knee pain. A total of 504 adolescents were successfully contacted and asked standardised questions in a telephone interview. ^6^ If they reported anterior knee pain with an non-traumatic onset, as opposed to traumatic onset, they were offered a clinical examination at the local hospital by an experienced rheumatologist to determine the specific knee condition. Two hundred and four adolescents were invited for a clinical examination and 180 accepted, 8 adolescents did not show up, leaving 172 adolescents who were examined. Of these, 153 were diagnosed with PFP, but 32 were subsequently excluded with the main reasons being the worst pain experienced the previous week being less than 30 mm on visual analogue scale (VAS) and patients with additional knee conditions (e.g. PFP combined with iliotibial band syndrome). Of the remaining 121 adolescents there were 106 who consented to contacting their GP (n=55 in total) and assess information regarding the period with patellofemoral pain prior to being enrolled in the APA-2011-cohort and a subsequent RCT. ^13^ These 106 were the focus of the current report and these data has not been published before or used in other reports.

Five GP’s never responded to our enquiry. Questionnaires containing information for 98 adolescents from their medical record were received from the remaining 50 GPs. The medical records were retrieved using the adolescent’s unique personal identification number. Of these, 3/98 adolescents had shifted to the current clinic shortly before the questionnaire was sent and it was not possible for the GP to obtain the record from the previous GP. Therefore, a total of 95 adolescents were included in the data analyses.

### Development of questionnaire

To obtain information from the GP, a questionnaire was developed and pilot-tested to ensure comprehensibility. The first version of the questionnaire was distributed among three GPs. The GPs were asked about comprehensibility and if there were questions that may be misinterpreted. After their feedback, a second version of the questionnaire was implemented and tested among two other GPs. Thereafter the questionnaire ^37^ was deemed comprehensible with a minimal risk of non-comprehension and misconception.

Content of the questionnaire:

• The dates for all primary consultations regarding knee pain
• If there were more than one contact regarding knee pain, did the record suggest it was about the same type of knee pain
• Localisation of knee pain
• Physical examination - observations
• Diagnosis
• If treatment was initiated and what type of treatment
• If the patient was referred to another health professional

To make it easier for the GP to respond, each question was followed by pre-defined response options. The question on the diagnosis for example contained the following response options: Patellofemoral pain, patella tendinopathy, fat pad impingement syndrome, Mb. Osgood Schlatter, iliotibial band syndrome, and other diagnoses. If they chose “other diagnoses” they were asked to describe the diagnosis in free text. The questionnaire is available online as extended data. ^37^

### Statistics

Demographics are presented as mean and standard deviation except for non-normally distributed data, which are presented as median and interquartile range. Number of contacts, diagnosis, treatments given, and referrals were described as n/total n and 95% confidence interval for the proportion. No statistical hypothesis testing was done.

## Results

### Demographics

The majority of the sample consisted of females (81%) with a median age of 17 ( Table 1) and a pain duration of more than three years ( Table 1).

**Table 1.** Demographics.

	All (n=95)
**Age**	17 (16-18)
**Gender (% females)**	81%
**Weight [kg]**	64.8 (11.5)
**Height [cm]**	172 (8.5)
**BMI**	21.7 (2.9)
Average pain duration (months) *	37.5 (21;60)
**Bilateral patellofemoral pain (n)**	75
**Previously treated for knee pain (n)**	25
**Pain medication for knee pain (n)**	18
Pain at rest (Visual Analogue Scale) *	13.5 (4;28)
Pain during activity (Visual Analogue Scale) *	49.5 (36;64)
Worst pain last week (Visual Analogue Scale) *	48.5 (35;65)

^*^ Median (interquartile range).

### Seeking medical care

**Figure 1.**
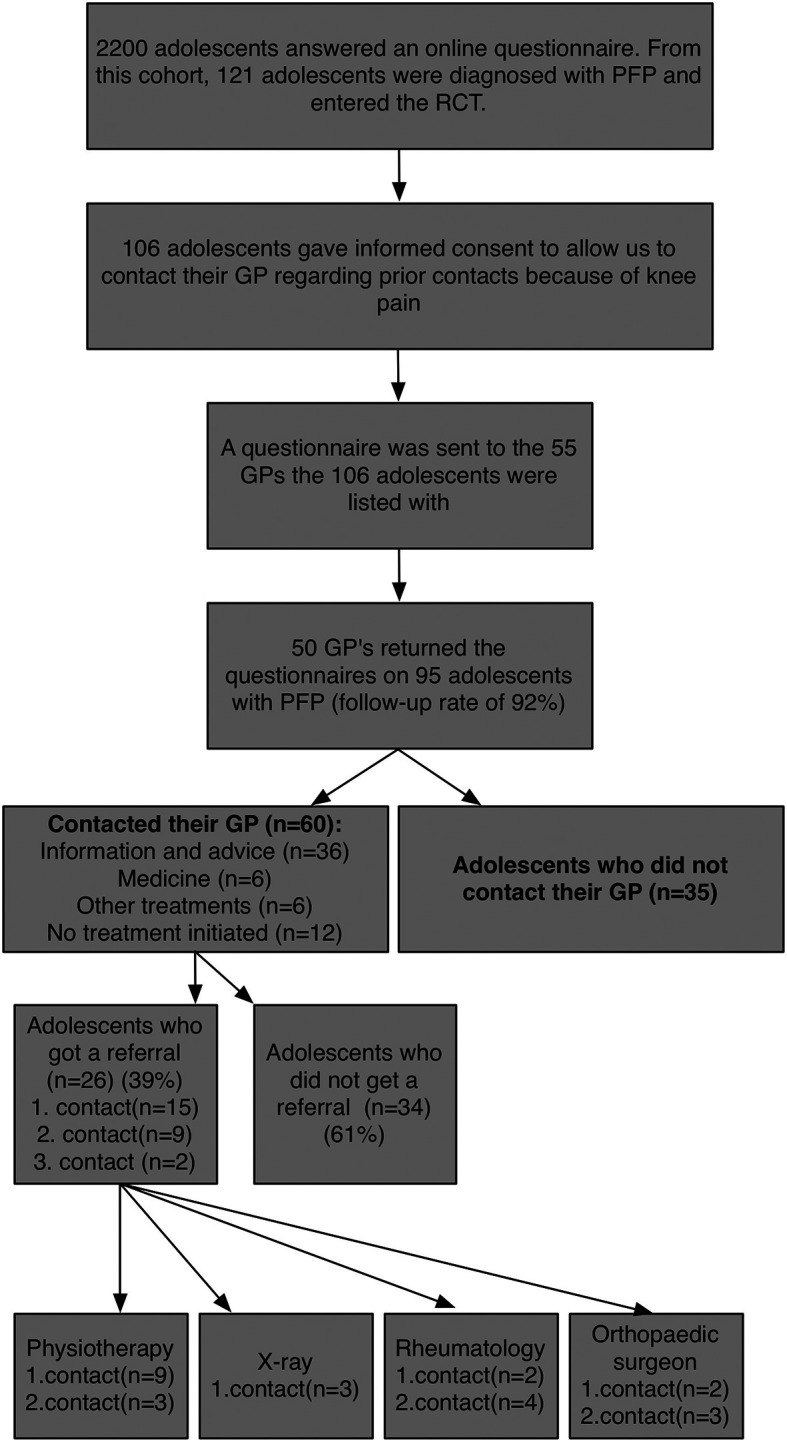
Flowchart.

The questionnaires based on the GPs medical record showed that 60/95 (0.63, 95%CI: 0.53-0.72) of the adolescents had sought medical care for their knee pain and 30/60 (0.50, 95%CI: 0.38-0.62) had consulted their GP more than once because of knee pain, Figure 1. Among those who consulted their GP more than once, the GP suspected the same knee condition in 8/30 (0.27, 95%CI: 0.14-0.45) adolescents. The median number of contacts was 1.5 (range 1-7).

There was good agreement between the information provided by adolescents regarding prior consultations and the information retrieved from the medical records at the GP. From the 60 adolescents who had consulted their GP because of knee pain, 50/60 (0.83, 95%CI: 0.72-0.91) reported having consulted their GP because of knee pain.

### Referral

26/60 (0.43, 95%CI: 0.32-0.56) of the adolescents who consulted their GP were subsequently referred. In Figure 1 the referrals are described (bottom line). Most frequently, they were referred to physiotherapy (n=11).

### Recommendation provided by the GP

In 12/60 cases (0.20, 95%CI: 0.12-0.32) (20%) no treatment was initiated by the GP, and from these, eight were later referred. The GP initiated treatment in 48/60 (0.80, 95%CI: 0.68-0.88) adolescents who contacted the GP. The most common recommendation provided was oral information and advice (36/48) (0.75, 95%CI: 0.61-0.85) followed by pain medication (6/48) (0.13, 95%CI: 0.05-0.30).

### Diagnoses and pain localisation

**Figure 2.**
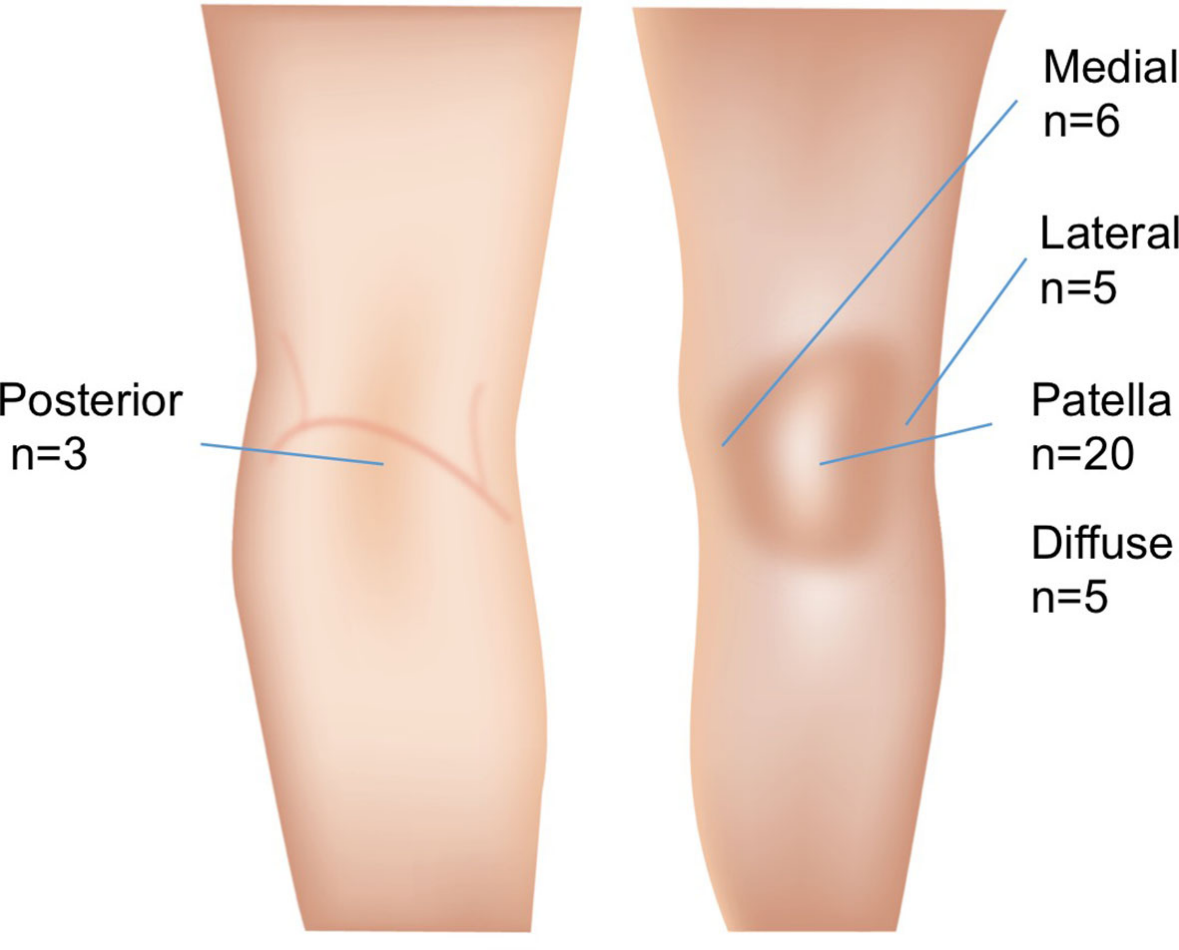
Pain localisation. 20 adolescents had pain isolated to the anterior aspect of the knee, 6 had medial knee pain, 5 had lateral knee pain, 11 had a combination of anterior and medial/lateral/posterior knee pain (not shown), 3 had posterior knee pain, 5 had diffuse knee pain without a specific location. In 10 of the adolescents there were no data on pain location available.

Data on diagnosis was obtained for 51 adolescents, Table 2 and Figure 2.

**Table 2.** Diagnosis from the general practitioner (GP) medical records.

Diagnosis in GP records	Number of adolescents
Patellofemoral pain	18
Patella tendinopathy	9
Possible meniscal injury	4
Distortion of the knee	4
Mb. Osgood Schlatter	4
Iliotibial band syndrome	3
Overuse of the knee	2
Possible anterior cruciate ligament injury	1
Other	6

### Objective findings

The most common objective findings were pain at palpation and swelling, Table 3.

**Table 3.** Objective findings from the general practitioners medical records.

Objective findings	Number of adolescents
Pain at palpation	17
Swelling	7
Patellofemoral crepitus	5
Pain during weight bearing, flexion or extension	4
Malalignment	3
Muscular problem (weakness or atrophy)	2
Psoriasis signs on knee	1
Suprapatellar tendinitis	1
Normal physical examination	13
Did not answer the question	7

## Discussion

This study showed that less than 2/3 of the adolescents with PFP consulted their GP because of knee pain. Among adolescents who consulted their GP, treatment was initiated by the GP in 48/60, while 26/60 at a later consultation were referred to primary or secondary care. These findings demonstrate the need for initiatives addressing both the adolescents and the GPs to ensure early standardised and evidence-based treatment of adolescent PFP.

### Seeking medical care

The percentage of adolescents who consulted their GP because of knee pain is similar to patients with generalised pain. Roth-Isiqkeit et al. (2005) reported that 51% of children and adolescents with pain had consulted their physician ^23^ while Perquin et al. (2000) reported that 57% had consulted their GP. ^24^ Masiero et al. (2010) reported that 74.2% of adolescents with musculoskeletal pain consulted a health provider, which in the majority of cases was a primary care practitioner, paediatrician or orthopaedist. ^25^ In the present study, the percentage (63%) who consulted their GP because of PFP pain as assessed by medical records is similar to our previous work on 504 adolescents with both traumatic and non-traumatic onset knee pain, where data were self-reported by participants (59%). ^6^ Previous studies reported that high pain intensity, long pain duration and older age were associated with seeking medical care among adolescents. ^6^^,^^24^ This is in line with the characteristics of our sample where the average age was 17, the average pain during activity was 49.5 out of 100 on the VAS and the average pain duration was 37.5 months. Therefore, an intriguing question is why so few adolescents consulted their GP? As outlined in the “Iceberg Theory of Disease”, not all people with medical symptoms decide to consult their GP. ^26^^,^^27^ There are several factors affecting the decision to consult the GP. These include the familial patterns of consultation (especially the mother providing advice for self-treatment), advice from friends, knowledge about the condition and psychological factors (i.e. stress, perceived vulnerability to illness, perceived severity of symptoms, perceived costs and benefits of action). ^26^^–^^28^ Previous studies also showed that the most common aetiology associated with seeking medical care for musculoskeletal pain was trauma. ^5^^,^^6^ Conversely, all adolescents with PFP in this study reported a non-traumatic onset of knee pain. It may be that adolescents with a non-traumatic onset of knee pain, which is a gradually evolving condition, are less likely to take their knee pain seriously or to act on their knee pain as opposed to those whose knee pain originates after a traumatic injury. ^6^ In addition, a subgroup of adolescents might decide to self-manage their knee pain with pain medication without consulting the GP. ^26^ In adolescents, higher use of pain medication might be associated with higher age and female gender, ^29^ in line with the demographic characteristics of our sample (mostly females in late adolescence). This is supported by our data, showing that 21% of adolescents used pain medication for their knee pain, ^13^ and also by figures of previous studies. ^23^^,^^30^ The tendency for self-medication behaviour is a potential issue, as evidence shows that adolescents have little knowledge on how pain medication works, potential side effects and often use pain medication inappropriately. ^31^^–^^33^

### Consultation frequency

Half of the adolescents, who initially consulted their GP, did not return for a second consultation. This could mean that their knee pain resolved after the treatment initiated by the GP. However, when the APA-cohort was initiated in September 2011, all 95 adolescents in the study had severe and long-lasting knee pain, which suggests that the knee pain did not resolve completely. The question is why the adolescents did not return to their GP and if they still had knee pain? One explanation is that they did not feel their knee pain was severe enough for the GP to be consulted and did not feel worried or anxious about their condition. ^26^ Another possibility is that the treatment initiated by the GP was unsuccessful (or the GP recommended to “wait and see”) and adolescents did not feel confidence in the GPs ability to handle their knee pain. ^26^ However, our data are in line with those of a recent primary care study which showed that only 45% of patients had two or more consultations and only 27.1% of patients with a persistent pain condition consulted 4 or more times. ^34^

### Diagnosis

Information on the GPs diagnosis illustrate the variety in medical diagnoses the adolescents received the first time they consulted their GP. Quite surprisingly only 18 out of 51 adolescents were diagnosed with PFP. The rest of the medical diagnoses were a combination of tendon related pain, Mb. Osgood Schlatter and iliotibial band syndrome. The pain localisation noted in the GPs medical records was in most cases compatible with PFP, however in several patients the pain localisation and physical findings points towards other knee conditions. This suggests that previous contacts to their GP represent separate knee conditions that may be precursors to PFP. Another possible explanation is that the quality of the diagnosis provided by the GP is too low to infer if their knee pain initially started out as a different knee condition than PFP.

### Action taken by the GP

To our knowledge, no one has previously investigated which treatments are most commonly used to treat adolescent PFP in primary and secondary care. Our previous study on self-reported treatment showed that of 504 adolescents with traumatic or non-traumatic onset knee pain, only 18% were currently receiving treatment and the most commonly received treatments were exercises and orthotics. ^6^ Among the 95 adolescents with PFP in the current study only 12 were referred to physiotherapy, which is the most common health professional to treat with exercises and orthotics. However, during the time period of 2006-2011 when our data was originally collected, only a few randomised trials existed and there was only weak evidence on the effect of exercises compared to information and advice. This, taken together with the variety of diagnosis given, suggests that adolescents were not treated in a standardized way at that time-point. Our data showed that 20% of adolescents (12/60) who consulted their GP were not initially given a treatment. This suggests that there is a small but considerable proportion of adolescents who are just initially told to “wait and see”. This recommendation might influence how adolescents interpret their symptoms (i.e. not severe enough for a treatment) and might affect their willingness to self-care as well as to seek treatment in case of their knee pain becoming more severe. Further studies in this area are needed to shed more light on this issue.

### Strengths and weaknesses of the study

The results from the current study may not be generalizable to all adolescents with PFP. As we enrolled adolescents who reported knee pain in September 2011, we did not include those who had been successfully treated by their GP for their knee pain. The retrospective cohort design is limited by the amount and quality of the information the GPs noted in the medical records. ^35^ However, because of the retrospective design the diagnosis, the choice of treatment and referral is unbiased as the GPs were not aware that their data would later be used for research purposes. ^36^ The sample size was fixed in advance, as the adolescents with PFP were participants in a randomized trial. The participants were, however, recruited from a population-based cohort. Larger studies may provide more precise estimates and be more generalizable.

This sample included adolescents from a population-based cohort who were all diagnosed with PFP by an experienced rheumatologist in September 2011. One of the strengths of the study is that Danish citizens have free and unlimited access to health care through a GP. Therefore, the care-seeking behaviour is not biased due to unequal access to health care. ^36^ However, our findings may not be generalizable to countries with unequal access to health care. ^37^ In addition, patients referred to physiotherapists have to pay about €20-30 per consultation. Therefore, economic considerations might have influenced whether or not patients wished to be referred to physiotherapy.

### Implications for treatment of adolescent patellofemoral pain

Adolescent PFP pain does not always have a favourable long-term prognosis. ^38^ A longer pain duration before initiation of treatment is associated with poorer long-term prognosis among patients with PFP ^20^ and early treatment is recommended compared to a “wait-and-see” approach. ^39^ There was a large heterogeneity in the clinical pathway and the types of treatments initiated in the sample of this current study, whose data originated from 10 years ago. Since that time, however, the Danish clinical guidelines for the treatment of adolescent knee pain have not been updated. Therefore, our study suggests that there is a need to develop clinical practice guidelines for treatment of adolescent knee pain as a first step to ensure evidence-based treatment which would improve the long-term prognosis.

## Conclusion

60 of the 95 adolescents diagnosed with PFP had previously consulted their GP because of knee pain. There was large heterogeneity among the treatment recommendations provided by the GP, the most common being general advice and information. However, there was also a substantial proportion (20%) of adolescents who were not initially provided a treatment. These findings demonstrate the need for initiatives addressing the GPs to ensure evidence-based treatment of adolescent PFP. As a first step, these initiatives should aim at establishing clinical practice guidelines for treatment of adolescent PFP.

## Data availability

### Underlying data

Harvard Dataverse: [Data regarding adolescents with patellofemoral pain] https://doi.org/10.7910/DVN/GW3JKL ^38^

The project contains the following underlying data:

- Clinical pathway data_anonymized_only english.xls (raw anonymized data from questionnaires)

### Extended data

Harvard Dataverse: [Data regarding adolescents with patellofemoral pain] https://doi.org/10.7910/DVN/GW3JKL ^38^

This project contains the following extended data:

- Questionnaire used for data collection.pdf

Data are available under the terms of the Creative Commons Zero “No rights reserved” data waiver (CC0 1.0 Public domain dedication).

## Software availability

There was no specific statistical code used for the descriptive analysis.

## Authors' contributions

All authors contributed to the development of the study purpose. MSR, CRR and MBJ collected data. All authors interpreted the results together. MSR, CRR and MBJ wrote the first draft of the manuscript and EMR, JLO, SR and AA gave feedback and helped revise the manuscript.

## Ethics approval

Ethical approval was obtained from the local ethics committee in the North Denmark Region (N-20110020).

## Consent to participate

All participants were required to give written informed consent for participation in the study.

## Consent for publication

All participants were required to give consent for publication together with the informed consent for participation in the study.
