# Supplementary material for: Care-seeking behaviour of adolescents with patellofemoral pain: a retrospective cohort study
Source: F1000Res. 2022 Feb 9;11:161. [Version 1] doi: 10.12688/f1000research.75667.1 (PMC10400934; doi:10.12688/f1000research.75667.1)
Supplement: Peer review history

# Peer review: Reviewer response for version 1

**DOI:** 10.5256/f1000research.79567.r180243

**Published:** 2023-08-03

**Authors:** Alicia Fernandez-Fernandez

**Affiliations:** 1 Nova Southeastern University, Fort Lauderdale, Florida, USA

This is a well-written manuscript that explores care provided to adolescents with PFP, including patterns of GP consultation as well as subsequent referrals and interventions. The findings confirm that there is a need for standardization of care protocols for adolescent knee pain in general practice, given that there was inherent variability in seeking care as well as in the subsequent care received.

 I have a few comments:

1. In the "seeking medical care section", this sentence is unclear: "From the 60 adolescents who had consulted their GP because of knee pain, 50/60 (0.83, 95%CI: 0.72-0.91) reported having consulted their GP because of knee pain." Note that the first part says 60 consulted the GP because of knee pain, but the second part says 50 did the same. Is there a typo or is the sentence just confusing? 
2. Was there any open-ended exploration of why in several cases no treatment was initiated by the GP, i.e.  do you have any insight into the rationale for these choices? Based on physical findings, level of pain, other aspects?
3. One of the limitations that you recognize is that original data collection was a long time ago - some (or all) of the data seems to be over 10 years old. How outdated do you think your findings are in terms of standardization of GP practices for adolescent knee pain?

Is the work clearly and accurately presented and does it cite the current literature?

Yes

If applicable, is the statistical analysis and its interpretation appropriate?

Not applicable

Are all the source data underlying the results available to ensure full reproducibility?

Yes

Is the study design appropriate and is the work technically sound?

Yes

Are the conclusions drawn adequately supported by the results?

Yes

Are sufficient details of methods and analysis provided to allow replication by others?

Yes

Reviewer Expertise:

Pediatric physical therapy, biomechanics, clinical decision-making

I confirm that I have read this submission and believe that I have an appropriate level of expertise to confirm that it is of an acceptable scientific standard.

# Peer review: Reviewer response for version 1

**DOI:** 10.5256/f1000research.79567.r171007

**Published:** 2023-05-15

**Authors:** Mitchell C. Selhorst

**Affiliations:** 1 Sports and Orthopedic Physical Therapy, Nationwide Children's Hospital, Columbus, OH, USA

This was a clear and well written manuscript. This article was on the care seeking nature of adolescents with PFP. This was a retrospective assessment of a PFP cohort. GP's of Adolescents with PFP who were previously seen were surveyed and a review of their medical chart was completed. A little over half were given education and advice by the GP. Most adolescents did not receive a referral for further care for their PFP. Based on the current understanding of PFP, a wait and see approach may not be best and a revamp of current AKP guidelines may be necessary.

**Specific questions and comments.**

- Table 1, it may be useful to readers to include the % of adolescents for B knee pain, previously treated and medication. I.e. B knee pain 75 (79%). Similarly for gender provide the n before the %.
- Please clarify those *“adolescents that did not contact their GP about their knee pain*”. Does this mean that they ended up in your larger research cohort without the GPs knowledge of knee pain, or does this mean that knee pain was reported on a routine healthy checkup with the GP? Often we see adolescents who reported pain at the wellcheck with GP but did not specifically contact the GP for that pain.
- In the section regarding Recommendation provided by the GP.
  In 12/60 cases no treatment was initiated by GP….eight were later referred. Does this mean that no treatment was initiated initially and subsequently the GP referred after pain continued, or that patient was referred on by another provider?

Is the work clearly and accurately presented and does it cite the current literature?

Yes

If applicable, is the statistical analysis and its interpretation appropriate?

Yes

Are all the source data underlying the results available to ensure full reproducibility?

Yes

Is the study design appropriate and is the work technically sound?

Yes

Are the conclusions drawn adequately supported by the results?

Yes

Are sufficient details of methods and analysis provided to allow replication by others?

Yes

Reviewer Expertise:

Physical Therapy rehabilitation of adolescents with Patellofemoral pain, and other overuse injuries

I confirm that I have read this submission and believe that I have an appropriate level of expertise to confirm that it is of an acceptable scientific standard.
